# Supplementary figures and images for: Rats spontaneously perceive global motion direction of drifting plaids
Source: PLoS Comput Biol. 2021 Sep 14;17(9):e1009415. doi: 10.1371/journal.pcbi.1009415 (PMC8462730; doi:10.1371/journal.pcbi.1009415)

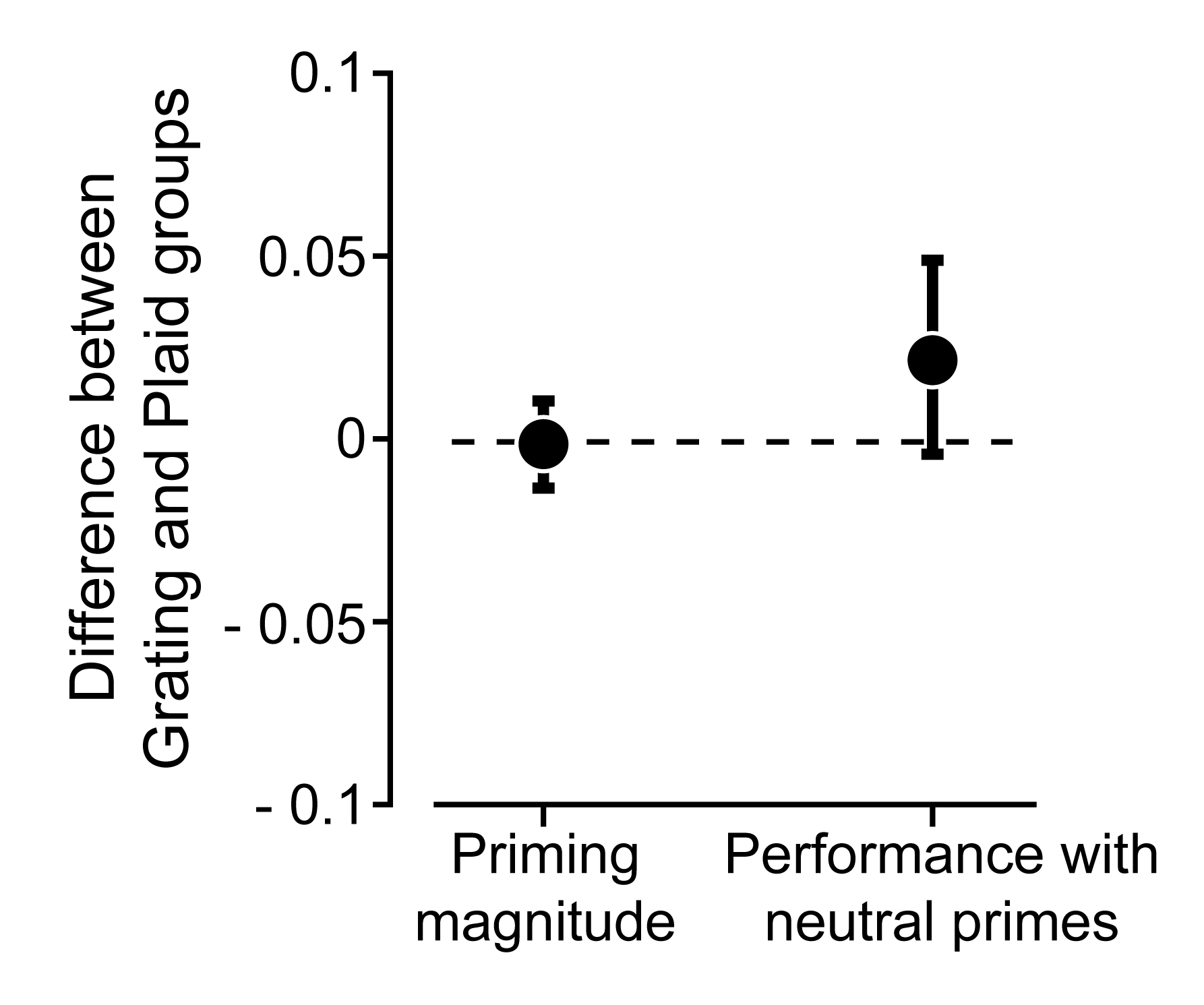

Supplement: S1 Fig — (A) Difference between the priming magnitudes measured for the G and P groups in the case of the identity-priming experiment (i.e., between the black bars in the insets of Fig 3A and 3B). Error bars are 95% confidence intervals obtained by bootstrap (see Materials and Methods). (B) Difference between the performances measured for the G and P groups with the neutral priming conditions, i.e., the 90°-drifitng (upward) graitng and plaid primes. Error bars are 95% confidence intervals obtained by bootstrap. (TIF) [file pcbi.1009415.s001.tif]

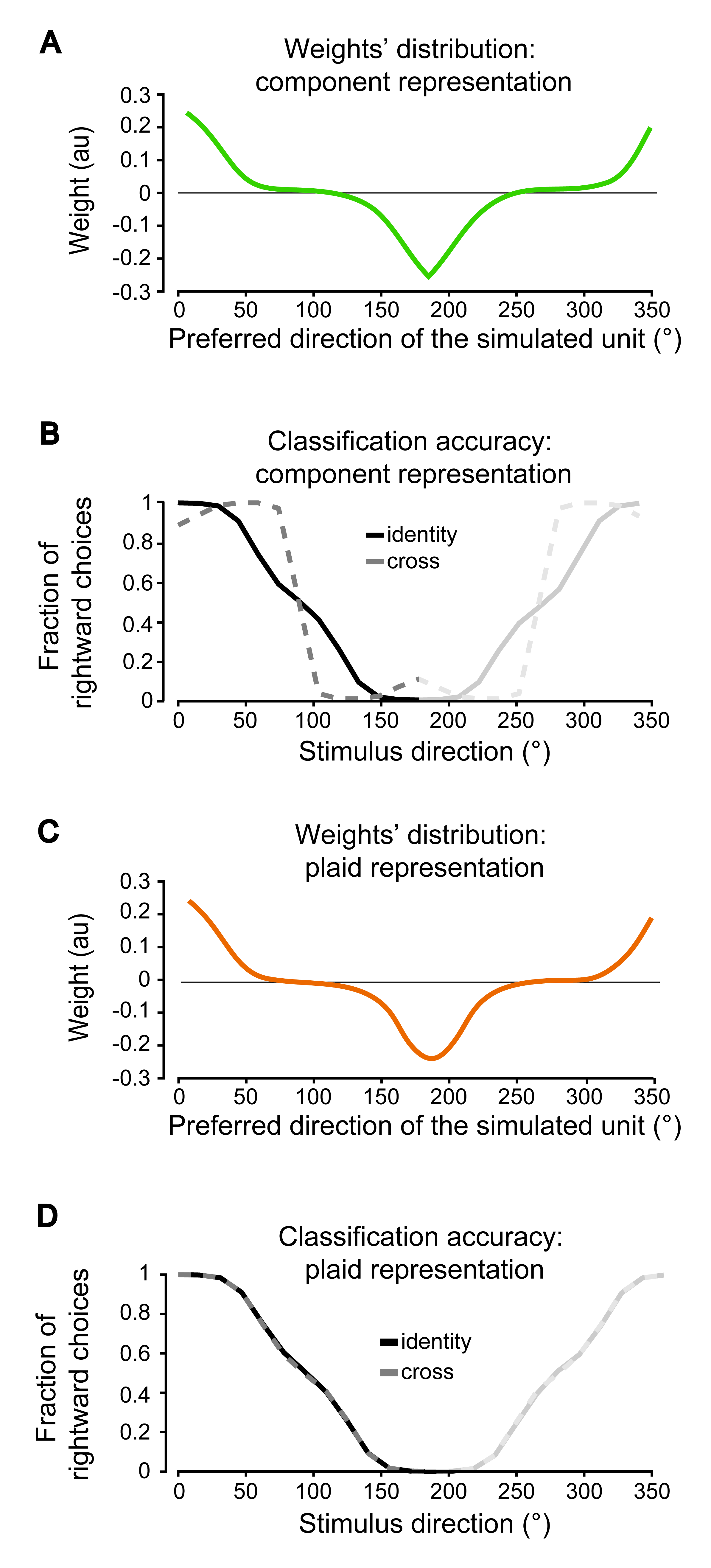

Supplement: S3 Fig — Same simulations as those shown in Fig 4C, 4D, 4E and 4F, but the classifier was trained to discriminate 0°- from 180°-drifting gratings rather than 0°- from 180°-drifting plaids. (TIF) [file pcbi.1009415.s003.tif]

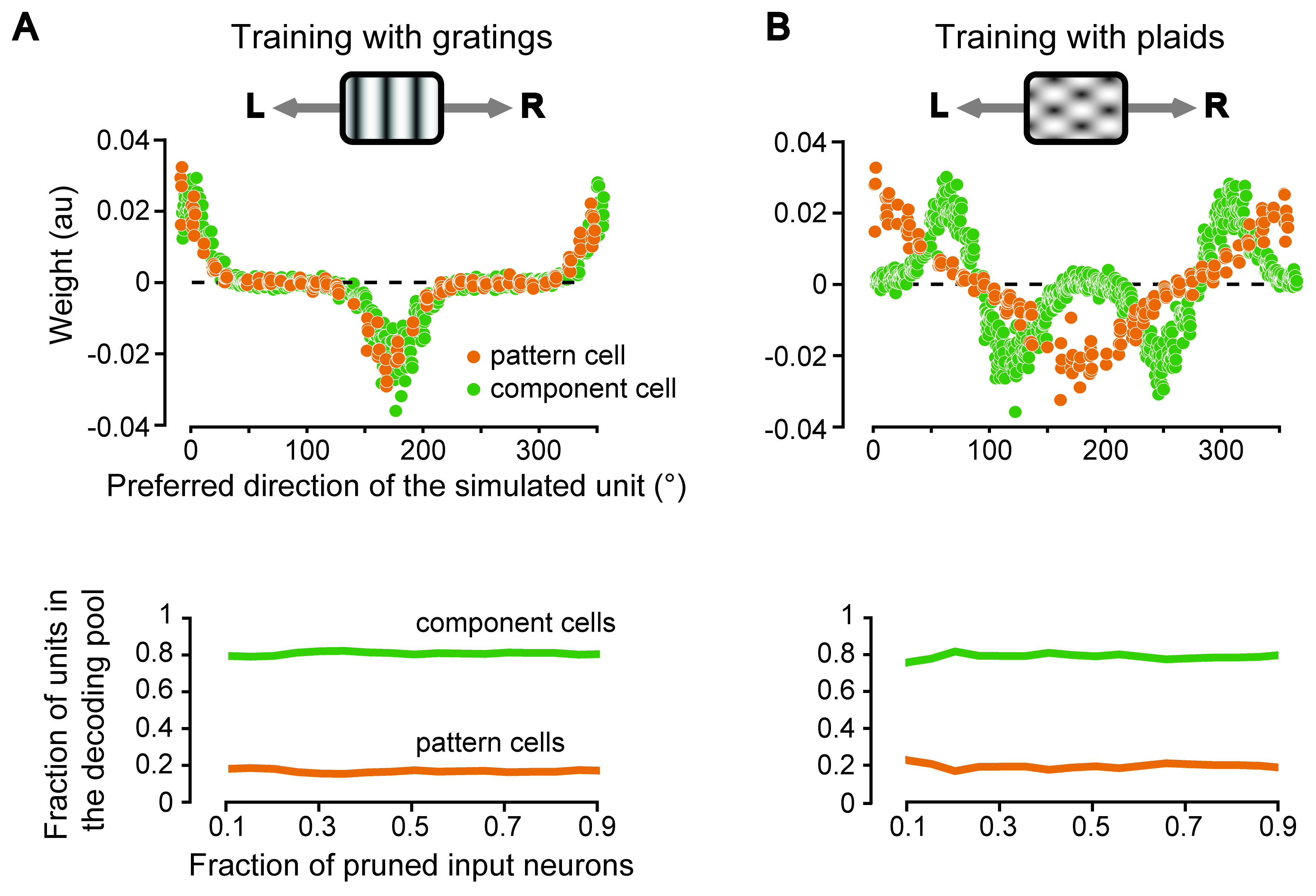

Supplement: S4 Fig — Same simulations as those shown in Fig 5C and 5D, but without imposing cross-orientation suppression in the component cells. (TIF) [file pcbi.1009415.s004.tif]
