# Supplementary material for: Rats spontaneously perceive global motion direction of drifting plaids
Source: PLoS Comput Biol. 2021 Sep 14;17(9):e1009415. doi: 10.1371/journal.pcbi.1009415 (PMC8462730; doi:10.1371/journal.pcbi.1009415)
Supplement: Attachment — Submitted filename: Mateucci_et_al_Response_to_Reviews.docx [file pcbi.1009415.s006.docx]

**Point-by-point response to the reviewers:**

**Plos Computational Biology manuscript PCOMPBIOL-D-21-00468**

We thank the reviewers for the overall appraisal of our manuscript, as well as for their comments and criticisms that were very helpful in improving our work.  Below, we address each reviewer comment (reported in blue/bold/italics font) and indicate where changes have been made in the revised version of the manuscript. For clarity, amendments to the manuscript are shown in red font.

**Reviewer #1**

***The original aim of this study was to test if the populations of neurons representing gratings and plaids are independent of each other. The authors found both. In grating trained rats, the additional plaid primer had no effect. In plaid trained rats the additional grating primer however had the same strong bias effect as the identical plaid stimulus primer.
It is however unclear what the authors were trying to prove. It is known from a large body of literature that pattern integration relies on component responses to gratings and pattern cells integrating these components into pattern responses. It is unclear how such an experiment should allow the dissection of these two populations.***

We are well aware that electrophysiology or imaging studies will be necessary to understand what neuronal populations support perception of local and global motion direction in rodents. However, in order to study the neuronal mechanisms underlying a given perceptual function, it is essential to characterize first that function at the behavioral level, so as to firmly establish its existence and properties. In the specific case of motion integration, despite previous physiology studies, it was not known whether rodents are capable of such advanced visual function at the level of perceptual behavior. In this context, the goal of our study was twofold. First, we wanted to test whether rats can generalize motion discrimination (spontaneously, without training) from gratings to plaids (and vice versa) – a necessary step to demonstrate motion integration at the perceptual level. Second, we wanted to explore the extent to which our behavioral data were consistent with: 1) the existence of pattern cells and component cells; and 2) the use of specific read-out mechanisms (i.e., the selection of specific neuronal populations by decision neurons). So, together our experiments and simulations try to achieve what the reviewer is asking: dissect the putative role of various populations of motion encoders and read-out mechanisms in supporting the observed motion discrimination behavior. In doing so, we believe they provide a useful roadmap for future neurophysiology experiments.

***It is in fact more obvious that the performance of the plaid trained rats is lower compared to those trained to discriminate gratings. Hence plaids are perceived less well. This might also explain why plaids have less of an effect as primers together with the better perceived gratings. It would have been clearer if the authors adjusted the stimuli to achieve similar performance, e.g. by using similar grey value distributions. Like this the results are very interesting but difficult to interpret.***

If we understand correctly, the reviewer is afraid that the failure of the plaids to produce a sizable priming on the perception of the gratings is due to the lower discriminability of the former – rat would perceive the plaids less well than the gratings and this would prevent the plaids from acting as affective primes for the gratings. This is certainly a reasonable concern, and this is why our experimental design was fully counter-balanced, so that we could measure rat sensitivity to both plaids and gratings when these stimuli were used either as targets or as primes. What is less clear to us is why the reviewer got the impression that rat performance with the plaids is lower than with the gratings. We assume that the reviewer reached this conclusion by comparing the identity priming curves/bars in our former Fig. 2 (now Fig. 3) and perhaps the performances with the 90º neutral prime stimulus on those curves. However, those curves, as well as the performances with the neutral primes, are very close for the two groups. In our previous version of the manuscript we did not compare them statistically, but we have done it now in our revision. The result of this comparison is shown in our **new supplementary figure (S1 Fig) and described in the manuscript at p. 11.** The difference between the amount of priming yielded by gratings and plaids in the identity priming condition for the two groups is statistically undistinguishable from zero (left dot) and the same applies to the difference between the priming magnitude produced by the neutral primes in the two groups (right dot).

These data however refer to the priming curves and not to the raw performances of the rats at discriminating gratings and plaids, which is the most relevant piece of information to address the reviewer’s concern. Such performances are available by looking at the data collected during the training phase of our study, when no prime stimuli were used and the rats of the two groups were simply trained to discriminate either gratings (G group) or plaids (P group). In our previous version of the manuscript we did not report any data from this phase of the experiment, because we wanted to focus on the priming experiment. Thanks to comment of the reviewer, we now realized that it is instead important to also show the data of the training phase.

These data are now shown in **our new Fig 2 and described in the manuscript at p. 8-9**. The figure reports both individual (panels A) and group average (panel B) learning curves for the rats of the two groups (i.e., the performance as a function of training sessions). As shown in the figure, learning followed a similar time course for both the G and P rats. As a result, the average number of training sessions that was required for the rats to reach the criterion performance of 70% (necessary to be moved to the priming experiment) was statistically the same for the two groups (about 25 sessions; see panel C). The same applies to the average discrimination performances reached by the two groups, which were again very close (81% vs. 79% correct for, respectively, the G and P group) and were not statistically different (see panel D).

We believe that the analysis of these new data fully addresses the concern of the reviewer by showing that the rats of the two groups were equally good at discriminating the motion direction of the stimuli they were trained with. In other words, our data show that rats are equally sensitive to plaid and grating motion direction. As such, the different cross-priming magnitude observed for the rats of the two groups cannot be explained by an overall lower discriminability of the plaid stimuli. We **explain and discuss this very carefully now at p. 21 of our revised manuscript**.

***In order to interpret their results the authors used different modeling approaches. First they simulated the discrimination accuracy afforded by component- and a pattern-based representations. Since component cells respond differently to gratings and plaids it is not surprising that populations of component cells also show different discrimination accuracy to these stimuli. And while pattern cell populations reliably show similar responses to gratings and plaids, they rely on input from component cells which’s not been included here.***

Thanks to this comment of the reviewer, we realized that we did not sufficiently motivate the need of carrying out the simulations shown in our previous Fig 3 (now Fig 4 in our revision). **We have now considerably expanded the part of the manuscript (see p. 14)** where we explain why it was important to check the consistency of the behavioral results of the P group with both a pattern- and a component-based representation.

Briefly, the failure of a component representation to support the cross- and identity-priming curve obtained for the P group is far from trivial for at least three reasons.

First, we are dealing with rodents, whose visual abilities are still largely unexplored, and which are capable of succeeding in apparently complex tasks using rather simple, low-level strategies. A good example is object recognition, where rats can rely on very simple, low-level cues (e.g., difference of luminance) in order to discriminate visual shapes (Minini and Jeffery, 2006). And the same applies, at the neurophysiology level, for V1 representations, which can support invariant recognition of visual objects across translation, scaling and rotation simply based on mean luminance differences (Tafazoli et al., 2017). Only by constraining the task/stimuli very tightly it was possible to reveal that rats can indeed recognize visual shapes using advance processing strategies.

Second, in the specific case of motion discrimination, very few studies exist on rodents. And when it comes to physiology, it is virtually unknown if rodents have anything like the functional pattern cells of monkeys. So, it is a real concern (that is necessary to rule out) the possibility that they might extract the global direction of the plaids using a much simpler, component-based strategy, thus only apparently achieving motion integration.

Finally, even in monkeys, component cells may actually display a patter-like behavior if direction tuning is broad enough (Tinsley et al., 2003). Again, it was important to rule out this possibility in our study, especially given that, in rodents, V1 neurons have slightly broader tuning curves than in monkeys. In fact, as shown by our simulations in our former S1 Fig (now S2 Fig), there is a parameter regime (k < 3) where similar identity and cross priming curves would be produced by a purely component based representation. As argued in our manuscript, rodents tuning curves are likely way sharper to possibly allow this to happen. But it was important to fully test this in our simulations, also to guide future experiments at the physiology level.

As a very final note to address the reviewer’s concern, it is true that we did not build pattern cells starting from component cells, because this was not the goal of our study. Other modeling studies have already shown that pattern cells can be obtained from component cells via proper non-linear integration mechanisms (Simoncelli and Heeger, 1998). So, there was no point in re-instantiating those models. We simulated ideal populations of component and pattern cells (knowing that the latter can be obtained mechanistically from the former) because our goal was not to investigate the mechanisms that can lead to the formation of pattern cells. Our goal was to test whether either a pattern- or a component-based representation (or both) was consistent with the behavioral results of the P group – and for that, we simply needed to simulate motion detectors with either an ideal component or an ideal pattern behavior.

***The second scenario is somewhat difficult to comprehend. The question is who tells the decision maker what drifting direction has been shown. The authors suggest „projection-
specific enrichment in the proportion of pattern cells relaying visual information to decision
making centers, such as posterior parietal cortex“. In this case one would expect pattern cells to be activated by plaid patterns should be better in guiding decision centers compared to activation by simple gratings.***

Thanks to this comment of the reviewer, we realized that we did not explain well enough why we simulated the second scenario. **We have now expanded the part of the manuscript where we introduce these simulations at p. 29 of our revised manuscript.**

Briefly, the simulated scenario is very similar to the one expected for the monkey dorsal stream. In monkeys, there is a clear hierarchy of processing of motion signals. In this hierarchy, V1 component cells encode local motion directions and they feed their responses to higher-order centers, like area MT. MT combines the inputs from V1 component cells so as to create pattern cells. In the most idealized description, these pattern cells can be seen as "shape invariant" motion detectors, which signal global motion direction regardless of the shape of the moving pattern/object. The rest of the brain then rely on these MT pattern cells to encode, represent and discriminate motion direction. The rest of the brain does not directly access the low-level representation provided by component cells in V1. Those cells only serve as building blocks to achieve MT pattern cells. Once the latter have been built, the rest of the brain forgets about V1 component cells and always relies on MT pattern cells, no matter whether the moving pattern to process is a grating, a plaid or any other visual pattern.

This is the scenario we simulated here. Hence, plaid stimuli would not be better than simple gratings to guide decision centers, via activation of the patter (MT-like) population. Because this population (by definition, in the ideal case) encodes equally well motion direction of gratings, plaids or any other stimulus. Only, more complex patterns, like plaids, may be more strongly affected by cross-orientation suppression. Thus, the decision units may compensate for this by assigning larger weights to (i.e., by making stronger synapses with) the pattern cells in case they are trained to discriminate the plaids (as shown in our simulations).

With regard to the question "who tells the decision maker what drifting direction has been shown", as in our previous simulations, the decision neuron learns in a supervised way to discriminate some specific motion directions (left vs. right) of some specific stimuli (plaids or gratings) during a training phase. Then it simply generalizes to any other possible visual inputs based on the weights it assigned to the units of the sensory representation at the time of training.

***Nevertheless the authors provide novel and interesting behavioral results that together with their simulation analysis provide predictions that can now be further tested experimentally. The manuscript however could be improved by clarifying the issues raised above.***

We thank the reviewer for this appraisal of our work. We took all their concerns very seriously and modified substantially our manuscript to better explain and motivate our simulations, with the inclusion of additional analyses on a part of the dataset that we originally did not present in our previous submission. We believe that this revision improved considerably our study.

***Minor points:***

***The plaid pattern shown in figure one looks more like 60 degrees difference in the two components.***

The fact is that one should look at the difference between the vectors indicating the directions of the constituent gratings of the plaid and not at the difference between the orientations of the grating themselves (which is by necessity 60º at two corners - the left/right ones - being 120º at the other two corners - the top/bottom ones). To make it clearer, **we have modified Fig. 1** by drawing the orientation of the constituent gratings (dashed gray lines) and the direction of their movement (black arrows). The figure makes it clear that the angle between the two vectors (shown in red) is 120º.

***It would be helpful to show the behavior setup. Where was the screen relative to threat? We’re the stimuli showstopper the binocular field infringing or above the animals nose? This might also be a difference to the cited rodent studies since neurophysiology studies usually place the monitor to the side of the mouse or rat.***

Since we already showed pictures of the behavioral rig in several of our previous studies (Zoccolan, 2015; Djurdjevic et al., 2018; Vanzella et al., 2019), we preferred not to show them again also in this manuscript. However, most of the details asked by the reviewers can be found in our description of the setup in the Methods. **We have further improved this section** by making it clear that rats faced the monitor frontally (differently from physiology studies, as the reviewer correctly guessed) – **see p. 33**. We also made explicit reference to the figures of three of our previous studies where pictures or CAD drawings of the setup are shown – **see p. 34**. From these images it can be understood very well the relative position between the rat and the various components of the rig (e.g., the stimulus display). Finally, **we have also provided an explanation of how the rig works at the beginning of the Results** – **see p. 6**.

**Reviewer #2**

***1) The MS would benefit from a more direct comparison between the models in Fig 3 and Fig 5 and the behavior in Fig 2. While the priming effect on behavior in Fig 2 is plotted as a function of the difference between cross priming and target directions, in the models, the difference between grating or plaids is analyzed as a function of direction only. Comparisons would be clearer if the accuracy of the classifier or fraction of rightward choices were compared for the stimulus for which the classifier in the models was trained vs the “cross primed” stimulus as a function of their angle difference like in the behavior in Fig 2.***

We understand the point of the reviewer. We also wondered whether it would have been more intuitive to plot the results of our simulations in the way the reviewer suggested (i.e., as a function of the angular difference between prime and target stimulus directions) to allow a more direct comparison with the plot of our former Fig. 2 (now Fig. 3). However, we realized that it was simpler and more intuitive to explain how the weight distribution and the classifier performance varied as a function of the stimulus direction. Once it is explained that the classifier (i.e., the simulated decision unit) is trained with the rightward (0º) and leftward (180º) directions and the fraction of correct choices is shown as a function of stimulus direction, we believe that our plot is easily understandable. In addition, we believe that, for completeness, is more instructive to show the whole range of weight distributions and performances around the circle, to emphasize the symmetrical nature of the learned classification. However, in our current revision, to ease the comparison with the behavioral data, **we have grayed out the right side of the plots showing the classifier performance** (**see our new Fig. 4D and F**).

***2) A model for the animals trained on gratings (G group) with no assumption of cross orientation or biased decision neurons is not directly discussed or included in Fig 3. While probably that model cannot explain the lack of cross priming effects of the G group (Fig 2A), it would be useful to discuss it to also get insights on the neural mechanism that the G group might be using to solve the task. If could also clarify why a different model for the G group might be needed to account for their small cross-priming effects as discussed in Fig 5.***

We agree with the reviewer: for completeness, it is better to repeat the simulations of our former Fig. 3 (now Fig. 4) also for the training with gratings. **We have now added a new supplementary figure (S3 Fig)** where we report the results of these simulations and **we have added an apposite section to the manuscript (see p. 19-20)** where we present and discuss these results. These new simulations show that while the identity priming curve of the G group is consistent with either a component- or a pattern-based representation (black line in S3 Fig), the lack of cross-priming cannot be explained by neither of them (gray lines). As the reviewer suggested, this is a nice way to introduce the rest of the simulations. It better motivates the need to assuming cross-orientation suppression and/or a reading-out mechanism that only focuses on pattern cells.

Inspired by this comment of the reviewer, **we have also included another supplementary figure (S4 Fig)** where we repeat the simulations of our former Fig. 4 (now Fig. 5) without imposing cross-orientation suppression on component cells. This is a control simulation that we already mentioned in our previous version of the manuscript, but **we now directly show the results and we better discuss the implications (see p. 28)**. That is, the lack of cross-orientation suppression would bring the decoding pool to be manly made of component cells, for both the G and P group. And this would be inconsistent with the behavioral data, because it would yield a sizable cross-priming, with a trend as the one shown in the other new supplementary figure (S3 Fig) that we added to address the concern of the reviewer.

***3) The statement in lines 83-93 of the introduction, is confusing. “Critically….such as posterior parietal cortex (PPC) “ . While it is certainly an issue that animals might associate reward with a feature of the stimulus that is different from the one intended by the experimenter, in the case of RDK experiments it is not clear what these features would be. This stimulus, unlike plaids, is designed so that there are no local cues that are sufficient for inferring motion direction and that global motion can only be extracted from spatial pooling. What features of RDK could animals have been associating in previous behavioral paradigms that were different from the intented ones? In my view these previous reports are consistent with the P group in this study, in the sense that they show that rodents can learn to report motion using only global cues. As it is written it seems to state that ambiguities of the features that rodent learn using RDK needed to be clarified and are addressed in the MS.***

We agree with the reviewer. The concern we raised in this paragraph applies to cases where an experimenter wants to test if two different classes of stimuli (e.g., drifting grating and plaids, or drifts gratings and RDKs) are spontaneously processed in the same way (e.g., by extracting from both kind of stimuli global motion direction). This is not the case, however, of the RDK studies we mentioned in our paragraph. **So, we fully changed this paragraph (see p. 5)**, emphasizing instead the differences between processing plaids and RDK stimuli (as derived from the monkey and mouse literature). Basically, the ability to extract global direction of RDK stimuli does not imply success in doing the same for plaid stimuli. RDK direction is way more easily processed by monkey MT neurons and by mouse V1 neurons. Hence the need of testing global motion integration with plaid stimuli in rodent species.

***4) Line 232 “ the neuronal population representing the direction of the gratings was largely inactive during the presentation of the plaids. “ . As no neuronal recordings were made in these experiments, this statement in the results sections is too speculative and misleading***

We only partially agree with this comment of the reviewer. It is certainly a speculative statement (and as such is presented in our manuscript) but we believe that it is not inappropriate to infer neural properties of stimulus representations from psychophysical experiments.  It is true that we did not record neuronal responses, but one of the goals of psychophysical studies is exactly to try inferring the properties of the underlying neural representation supporting the perceptual task under exam. Priming and adaptation studies are specifically meant to do so, because they measure the extent to which different stimuli (i.e., the primer and the target or the adapter and the target) are spontaneously perceived as similar by an observer. Absence of priming or absence of adaptation aftereffect can be safely interpreted as a signature that the neuronal populations encoding the two kinds of stimuli are different, or minimally overlapping, or at least very strongly activated by only one of the stimuli. **We have nevertheless softened our statement** that now reads "This inability of the plaids (when used as prime stimuli) to affect the discrimination (hence the perception) of the target gratings suggests that, for the rats of the G group, the neuronal population representing the direction of the gratings was poorly activated by the presentation of the plaids." – **see p. 13**
